# Dendritic Cells: Multifunctional Roles in Host Defenses to *Cryptococcus* Infections

**DOI:** 10.3390/jof9111050

**Published:** 2023-10-26

**Authors:** Kristie D. Goughenour, Ayesha S. Nair, Jintao Xu, Michal A. Olszewski, Karen L. Wozniak

**Affiliations:** 1 Research Service, Department of Veterans Affairs Health System, Ann Arbor VA Healthcare System, Ann Arbor, MI 48105, USA; 2 Division of Pulmonary and Critical Care Medicine, Department of Internal Medicine, University of Michigan Health System, Ann Arbor, MI 48109, USA; 3 Department of Microbiology and Molecular Genetics, Oklahoma State University, Stillwater, OK 74078, USA

**Keywords:** dendritic cells, cryptococcus, fungi, immunity

## Abstract

Fungal infections are an increasingly growing public health concern, and *Cryptococcus* is one of the most problematic fungal organisms causing substantial mortality and morbidity worldwide. Clinically, this high incidence of cryptococcosis is most commonly seen in immunocompromised patients, especially those who lack an adaptive T cell response, such as HIV/AIDS patients. However, patients with other underlying immunodeficiencies are also at an increased risk for cryptococcosis. The adaptive immune response, in particular the Th1/Th17 T-cell-mediated responses, to pulmonary *Cryptococcus* infections are required for host protection. Dendritic cells (DCs), encompassing multiple subsets identified to date, are recognized as the major professional antigen-presenting cell (APC) subset essential for the initiation and execution of T-cell immunity. Apart from their prominent role in orchestration of the adaptive arm of the immune defenses, DCs are fully armed cells from the innate immune system capable of the recognition, uptake, and killing of the fungal cells. Thus, DCs serve as a critical point for the endpoint outcomes of either fungal control or unrestrained fungal infection. Multiple studies have shown that DCs are required for anti-cryptococcal defense in the lungs. In addition, the role of DCs in *Cryptococcus gattii* infections is just starting to be elucidated. *C. gattii* has recently risen to prominence with multiple outbreaks in the US and Canada, demonstrating increased virulence in non-immunocompromised individuals. *C. gattii* infection fails to generate an inflammatory immune response or a protective Th1/Th17 T cell response, at least in part, through a lack of proper DC function. Here we summarize the multiple roles of DCs, including subsets of DCs in both mouse and human models, the roles of DCs during cryptococcal infection, and mechanisms by cryptococcal cells to attempt to undermine these host defenses.

## 1. Introduction

Cryptococcosis is an infectious disease caused by *Cryptococcus-*species clusters that have been subsequently subdivided into several species including *C. neoformans*, *C. deneoformans*, and *C. gattii*, primarily affecting immunocompromised hosts with different levels of immunosuppression [1]. These fungal pathogens cause invasive infections with a high mortality rate, and survivors have lasting complications such as neurologic deficits as a result of central nervous system (CNS) damage [2]. Dendritic cells (DCs) shape adaptive immune responses by capturing the antigens and presenting the processed antigens to naïve T cells [3]. Compared to other immune cells, DCs show the greatest potential for T cell activation [4,5], serving as the most potent antigen-presenting cells and a bridge between the innate and adaptive immune responses. Upon infection, DCs can mature (increased surface expression of MHC II and costimulatory molecules) and begin to migrate to the draining lymph nodes to interact with naïve T cells, thereby initiating the adaptive immune response. However, only a small fraction of DCs recruited for the infected organ migrate to the regional nodes [6,7,8], suggesting that the majority of DCs act locally, e.g., restimulating the antigen-experienced T cells to produce required cytokines at the site of infection, and activate fungal killing directly or in collaboration with other cell types. In this context, lung DCs serve as an important distal effector cell capable of activating T cells and performing effective fungal killing. Here we focus on the current understanding of the interaction between the fungal pathogens and DCs, DC subsets, and their functional diversity.

### Dendritic Cell Subsets

**Figure 1:**
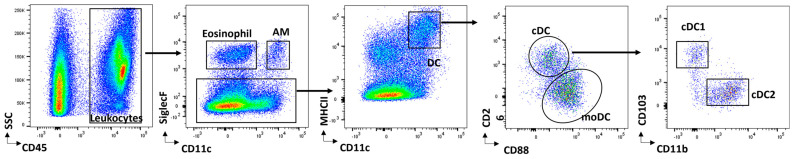
DC gating strategies. Flow cytometry gating strategy used for analysis or sorting of DC subsets during cryptococcal infections in mice. C57BL/6 mice were infected intratracheally with *C. deneoformans* strain 52D. The lungs were enzymatically dispersed, and the leukocytes were isolated and stained for flow cytometry. The gating steps and antibody markers used to gate on monocyte-derived, and conventional DC subsets are shown at each plot.

DCs are a heterogeneous group of innate immune cells that function in surveillance throughout the body [9,10]. The heterogeneity of the DC population is derived from the ontogeny of these cell subsets from different sources [10,11,12], and the nomenclature used is dependent on its species, location, phenotype (including cell surface markers), and immunological function. Mouse DC subsets encompass CD11b^+^ conventional pulmonary DCs (cDC2), CD103^+^ conventional pulmonary DCs (cDC1), plasmacytoid DCs (pDCs), Langerhans DCs (LCs), and inflammatory monocyte-derived DCs (MoDCs) [11,13,14,15,16,17] (Table 1 and Figure 1). Specifically in the naïve murine lung, the resident DC populations include the CD11b^+^ conventional DCs (cDC2) and CD103^+^ conventional DCs (cDC1) as well as a very small population of pDCs [18,19]. In humans, circulating DC subsets containing DC precursors include classical monocytes, non-classical monocytes, and intermediate monocytes, while pulmonary subsets include CD1c^+^ CD14^−^ DCs, CD1c^+^ CD14^+^ DCs, langerin^+^ (CD207^+^) DCs, and pDCs (Table 2) [14,20,21,22,23,24].

Several studies in the mouse model have shown that cDC2 cells migrate to the lungs in response to cryptococcal infection [29,30]. However, these cells are also residents of the naïve mouse lung [18]. These cells can induce protective immune responses against the fungal pathogen. cDC2s help in clearing the pathogen from the lungs, and this subset, along with cDC1s, generate memory-like immune responses towards the fungal pathogen [31]. Like cDC1s and cDC2s, MoDCs avidly ingest *Cryptococcus*, and, upon IFN-γ activation, upregulate the set of classical activation markers (iNOS, IL-12b, and MHCII) and antifungal mechanisms [32]. Whether pulmonary-recruited MoDCs subsequently migrate to the draining nodes or remain in the lungs as distal effector cells is under investigation. The pDCs represent a distinct class of DCs differentiated from murine bone marrow. They display anti-cryptococcal activity in vitro, which relies on the use of reactive oxygen species (ROS) to clear the organism [33]. In studies examining the ex-vivo role of resident lung DC subsets from naive mice derived from male mice, the cDC2s allowed significant intracellular cryptococcal replication, while cDC1s had antifungal activity. Interestingly, these two conventional DC subsets derived from female mice both had antifungal activity [18]. The data from humans are more limited and pertain to pulmonary DCs present in the healthy human lung. These studies led to the identification of DC subsets with the help of transcriptional profiling and flow cytometry [21]. Recent studies have shown that CD1c^+^ CD14^−^ and CD1c^+^ CD14^+^ human conventional pulmonary DC subsets interact differently with *C. neoformans* in terms of cryptococcal uptake, but each is able to efficiently kill *C. neoformans* [13].

## 2. Functional Capacities of DCs

### 2.1. Recognition

The initial mobilization of host defenses against pathogens is triggered by the recognition of pathogen-associated molecular patterns (PAMPs) by specialized pattern recognition receptors (PRRs) that recognize these PAMPs. DCs were identified as the cell subset that abundantly expresses multiple classes of PRRs, of which many serve to recognize fungal components. Below, we will predominantly focus on the roles of toll-like receptors (TLRs) and c-type lectin receptors (CLRs), as their roles in *Cryptococcus* recognition by DCs have been characterized best.

TLRs are highly expressed by DCs, leading to the recognition of patterns expressed by a broad range of viral, bacterial, and fungal pathogens [34,35,36,37,38,39,40,41]. While TLR2 and TLR4 can be activated by cryptococcal components [42], these receptors do not play major roles in the defense against cryptococcal infections [43,44]. TLR2^−/−^ and TLR-4^−/−^ mice infected with *C. neoformans* show normal pro-inflammatory cytokine responses and similar survival rates to their wild type counterparts [43,44], and there are no reports of cryptococcal infections associated with their deficiencies. However, a more profound effect resulted from the deletion of the adaptor molecule MyD88 [44], which is central in the signaling of multiple TLRs, including TLR2, TLR4 and TLR9, and the IL-1 receptor. Interestingly, MyD88 plays a role in fungal control in the lungs and the survival of mice even though its loss does not have an effect on protective cytokine levels in the lungs [44]. Recent evidence in macrophages shows that TLR4 is involved in down-regulating non-opsonic phagocytosis of *Cryptococcus*. In TLR4 KO macrophages, phagocytosis is increased, which depends on the scavenger receptor MSR1 recognizing oxidized lipids [45]. The intracellular TLR9 receptor has a documented role in the detection of *Cryptococcus*. Unmethylated CpG DNA from *C. neoformans* stimulates BMDCs to become activated [46]. TLR9^−/−^ mice (and MyD88^−/−^, which is also downstream of TLR9 signaling) have severely reduced DC activation in response to unmethylated CpG cryptococcal DNA [46]. In addition, BMDCs have been shown to detect URA5 gene fragments from *Cryptococcus* via TLR9 to induce IL-12p40 production [47]. TLR9 signaling contributes to pulmonary accumulation and the activation of DCs with MHCII, CD40, and CD80 upregulation through induction of CCL7 [48]. TLR9^−/−^ mice are more susceptible to pulmonary infection and have reduced fungal control in the lungs and spleen, and show impaired pulmonary lymphocyte recruitment during the adaptive phase of the immune response [46,49]. A similar importance of TLR9 is seen in *C. gattii* infections. TLR9^−/−^ mice infected intratracheally with *C. gattii* are more susceptible to infection and have higher fungal burdens in their brains and spleens, organs associated with dissemination [50]. Interestingly these mice can control fungal growth in the lungs even though the lungs of the TLR9^−/−^ mice have lower levels of IFN-γ and IL-17A [50].

Another well-established family of receptors contributing to the detection of fungal pathogens by DCs are CLRs, including Dectin-1, Dectin-2, Dectin-3, Mincle, and the mannose receptor (MR, CD206) [51]. The CLRs recognize carbohydrate moieties present in the cell walls of fungal organisms. The role of these receptors in cryptococcal detection by DCs is not very clear, possibly due to redundancies or multi-receptor collaboration. Dectin-1, which recognizes β-glucans, appears to be dispensable for cryptococcal infections in a pulmonary murine model of infection, and BMDCs from Dectin-1 KO mice generated similar levels of IL-12p40 upon cryptococcal co-culture compared to WT BMDCs [52]. Dectin-3, another CLR, is able to recognize α-mannans found in the cell walls of fungal pathogens [53]. However, recent studies show that Dectin-3 is not necessary for stimulating protective immune responses against *C. neoformans* infection. Murine studies comparing Dectin-3-deficient and wild type mice did not show any significant differences in the anti-cryptococcal activity of Dectin-3^−/−^ cDC2s or BMDCs [54]. However, in pDCs, Dectin-3 appears to be required for anticryptococcal activity [33]. Dectin-2, another CLR, recognizes mannose polysaccharides, thereby stimulating an immune response against fungal pathogens [55]. The ligands with mannose moieties present on the cell walls of the fungal pathogen activate the DCs once they are stimulated with *C. neoformans* [56]. Dectin-2 deficiency enhanced the expression of Th2 cytokines and mucin production during cryptococcal infection, but it simultaneously did not have any effect on Th1 cytokines and fungal clearance [57]. Multiple studies have also shown that BMDCs from Dectin-2 KO mice have reduced the production of IL-12p40 and TNF-α, as well as reduced the expression of CD86 and MHC-II [56,57]. However, the final impact of virulence is complicated. While mice infected with cryptococcal spores show no reduced survival in Dectin-2 KO mice [58], when interactions with the cryptococcal yeast phase are examined, Dectin-2 plays a role in phagocytosis and is mediated through CARD9 signaling involving Syk and P13K [59]. Thus, the role of Dectin-2 signaling in DC–cryptococcal interactions appears to be contextual and based on the state of the cell wall. Reports of *C. gattii* growth media conditions altering Dectin-2 signaling support this idea [60], and studies show that when *C. neoformans* was treated with liposomes delivering Amphotericin-B and soluble Dectin-2, the Dectin-2 targeting of the liposome packaged drug had an improved fungicidal effect [61].

Glycoproteins have been known to stimulate T cell responses against a wide variety of antigens. Mannose residues, which are the most common form of carbohydrate that is used by fungi for the synthesis of glycan, are usually present on the fungal glycoproteins [62]. Another c-type lectin receptor, the mannose receptor (MR) (in addition to the Fcγ receptor II), is required for antigen presentation by DCs to T cells in cryptococcal infections [63]. Highly mannosylated proteins from the cryptococcal cell wall, called mannoproteins (MP) [64,65] have been shown to induce DCs to produce pro-inflammatory/Th1 cytokines, such as TNF-α and IFN-γ [66,67]. MP98 is able to induce DC maturation, stimulate T cells efficiently and promote CD4 and CD8 T cell proliferation via cross-presentation [68,69,70]. Studies suggest that DCs act as the bridge between innate and adaptive immune responses through a process involving the recognition of cryptococcal MP98 by the mannose receptor (MR) [69]. MP98 binds to the MR (CD206) on DCs [71], although CD209 also has some affinity [69]. MR KO mice have increased mortality and higher fungal burdens in the lungs during pulmonary infection compared to WT mice [71]. Interestingly, in vitro DCs from MR KO mice had similar uptake of MP compared to WT mice, and neither WT nor MR KO DCs upregulated maturation markers (MHCII, CD80 or CD40) upon MR stimulation by MP98 [71]. Studies also suggest that if the MR and the TLRs are stimulated cooperatively, it would enhance the DC response to mannosylated antigens [68].

Several other immune receptors are also important in controlling cryptococcal infections in the lungs. The complement receptor CR3 plays an important role in the in vitro phagocytosis of unencapsulated *C. neoformans* [72]. The scavenger receptor MARCO is required for early fungal control, as MARCO KO mice fail to properly recruit MoDCs, which also fail to properly mature and activate [73]. In summary, multiple known PRRs capable of fungal PAMP recognition are present on DCs and contribute to cryptococcal antigen sensing. However, the existence of additional receptors and signaling pathways for cryptococcal detection by DCs is likely.

### 2.2. Initial Degradation and Processing of Cryptococcus by DCs

**Figure 2:**
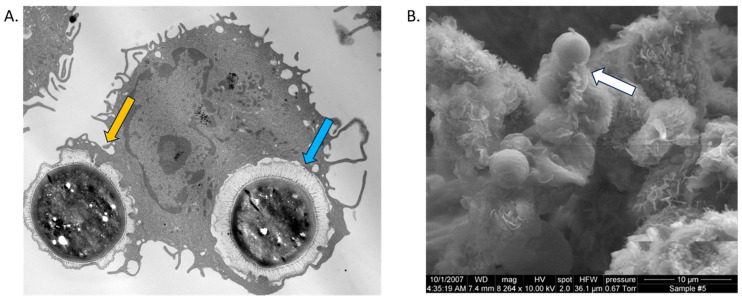
Phagocytosis of *Cryptococcus* by DCs. Electron microscopy (EM) images of zipper-type phagocytosis by BMDCs from BALB/c mice of opsonized *C. neoformans* at 30 min post-incubation. (**A**) Transmission EM (TEM) showing one internalized *C. neoformans* (blue arrow) and another being ingested by symmetrical pseudopods (orange arrow). (**B**) Scanning EM (SEM) showing DCs with symmetrical pseudopods during phagocytosis (white arrow).

**Figure 3:**
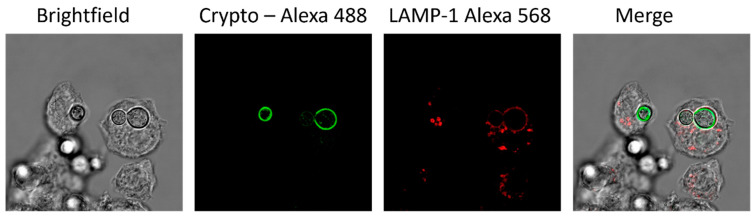
*Cryptococcus* localizes to the lysosome following phagocytosis. BMDCs were differentiated from BALB/c mice. Images show localization of *C. neoformans* to lysosomes following opsonization and phagocytosis at 20 min post-incubation. Confocal images show brightfield, *C. neoformans* stained with Alexa 488 succinimidyl ester (green), LAMP-1 stained with anti-LAMP-1 antibodies (Alexa 568) (red), and the merged image (Magnification 63×).

DCs possess phagocytic and complement receptors that enable them to phagocytose and kill pathogens along with their antigen presentation capabilities [63,74]. In vitro studies focusing on murine BMDCs and human PBMC-derived DCs showed that DCs exhibited maximal binding, uptake, and antifungal activity against *C. neoformans* opsonized with anti-capsular antibody or complement [75,76] (Figure 2). Following conventional zipper-type phagocytosis by DCs, phagosomes fuse with lysosomal compartments. These compartments acquire lysosomal-associated membrane protein 1 (LAMP-1) (Figure 3) [76] and require acidification to recruit tetraspanin CD63 to *C. neoformans-*containing phagosomes [77]. Following acidification, the cryptococcal cells are degraded by a variety of oxidative mechanisms [13,20,33,74] as well as non-oxidative mechanisms including lysosomal enzymes, such as cathepsin B, Coronin, HNE, NOSTRIN, MMP25, and MPO [13,76,78]. How *C. neoformans* affects the lysosomal maturation pathway is not well understood in DCs, but early studies in macrophages show *C. neoformans* does not prevent phagolysosome fusion and can survive in an acidic environment [79,80]. However, recent studies, mainly in monocytes and macrophages, have found that *C. neoformans* is capable of modulating the phagosome’s pH and development [77,81,82,83,84].

### 2.3. Antigen Presenting in Lymphoid Tissues to Prime T Cells: Roles of cDCs

**Figure 4:**
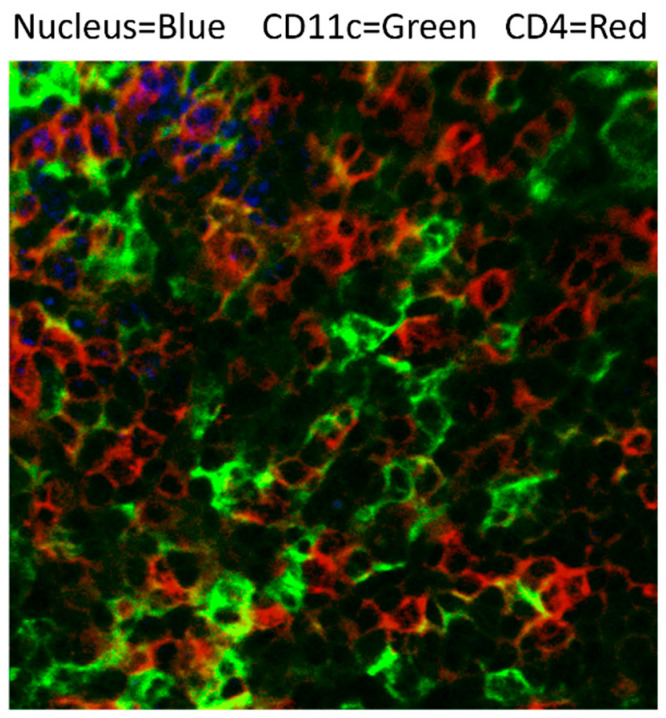
DCs directly interact with T cells in draining lymph nodes. Interactions of DCs with the expanding CD4 T-cells in the lung-draining lymph nodes (LDLN) during cryptococcal infections in mice. CBA/J mice were infected intratracheally with *C. deneoformans* strain 52D. The expanding lung-draining nodes were harvested at 14 days post-infection and frozen in media to perform fresh-frozen sections followed by antibody staining and confocal imaging. Note the abundance of DC recruited into the nodes and the high frequency of direct interactions between DCs and CD4 T-cells in the expanding T-cell zone (Magnification 40×).

Following cryptococcal degradation, DCs play an essential role in inducing both protective and non-protective cell-mediated responses against cryptococcal species by presenting antigens to T cells and inducing T cell proliferation to cryptococcal antigens [29]. DCs are the most efficient cells in the presentation of cryptococcal antigen to naïve T cells, and are required for the generation of a protective T cell response [63,69]. Since lungs are the portal entry for cryptococcal infection, the initial antigenic selection of T cells, their expansion, and pre-polarization occurs predominantly in the lung-associated lymph node (LALN) and these processes are orchestrated by DCs [85,86,87,88] (Figure 4); other secondary lymphoid organs are also likely to be involved [89,90,91,92]. T cell expansion during cryptococcal infection was completely abolished in CD11c-cre MHCII fl/fl mice [93]. Furthermore, mice deficient in cDC2s, abrogated using CD11c-cre IRF4 fl/fl mice, experienced blunted Th2 cell accumulation with cryptococcal infection [93], while BATF3^−/−^ (a knockout of cDC1s [94]), and CCR2^−/−^ (a knockout of monocytes and MoDCs) [95] mice replaced protective Th1 with robust Th2 responses during cryptococcal infection [27,93]. There are multiple mechanisms contributing to Th1/Th17 polarization during pulmonary cryptococcal infection, and they continue to be investigated. It has been shown that MP can stimulate lymphoproliferation in CD4^+^ T cells in the peripheral lymph nodes of WT infected mice, but not in MR KO mice [71]. The analysis of the draining lymph nodes [LCs, cDC1/myeloid DCs (MDC) and lymphoid DCs (LDC) in three mouse DC subsets showed that, in a protective immune response, Langerhans cells and MDCs are required; whereas, higher levels of LDCs indicate a nonprotective immune response and may serve as negative regulators [90]. Proper accumulation of DCs (Langerhans cells, MDCs, and LDCs) in the draining lymph nodes of mice generate an anti-cryptococcal response which is heavily reliant on TNF-α [89,96]. The loss of TNF-α also alters the cytokines produced by the DCs that migrate to the LALN from Th1/Th17 polarizing (reduced IL-12, TNF-α, IL-23 and IL-21) to Th2 polarizing, resulting in impaired T cell responses and a lack of fungal control in the lungs [85]. This non-protective response in TNF-α depleted mice is similar to what is seen if cryptococcal antigen-pulsed immature BMDCs are used to stimulate a T cell response [96]. In further support of this, an engineered *C. neoformans* strain expressing murine TNF-α is able to improve fungal control by enhancing activation of cDC1 cells [97]. This well-established, protective effect of TNF-α in cryptococcal infections has recently been shown to involve TNF-α preprogrammed DC precursor cells to generate a protective response [32]. Pulmonary infection with *C. neoformans,* engineered to produce murine IFN-γ, also increases the infiltration of cDC2 cells to the lung during a protective immune response [30]. DCs that undergo maturation with TNF-α become stably polarized to a classical, protective DC1 response, even upon subsequent alternative activation stimulation (by IL-4 or heat killed *Cryptococcus*) [32]. This is achieved through increased H3K4me3 association to the promoter sites of DC1 characteristic genes such as the IL12β and NOS2 genes [32]. These TNF-α preprogrammed DCs generate a protective Th1 polarized CD4^+^ T cell response during infection [32].

### 2.4. Restimulation of T Cells

In pulmonary cryptococcal infections, the full activation and polarization of T cells is not complete in the lymphatic tissue. Few CD4^+^ and CD8^+^ T cells expressing an activated phenotype are detectable in the lymph nodes, spleen, or blood [87]. Instead, re-stimulation in the lungs of mice is required to generate the high frequencies of activated T cells seen in the lungs and for their terminal polarization to IFN-γ producing effector cells [87]. Although details are still under active research, CCR2 dependent MoDCs have been regarded to present antigens in tissues to boost effector functions of newly recruited T cells or to rapidly activate tissue-resident memory T cells [98]. CCR2 is required for the recruitment of cDC2 cells to the lungs, thereby generating the nonprotective Th2 response in CCR2^−/−^ mice [27,99]. This non-protective response is a result of a lack of CD4^+^ T cell re-stimulation in the lungs. CCR2 KO mice fail to recruit enough cDC2s to the bronchovascular infiltrates, which represent tertiary lymphoid tissue [27]. In WT mice, the co-localization of MoDCs and CD4^+^ T cells in these bronchovascular infiltrates generate an increased expression of protective IL-12 and IFN-γ, while in CCR2 KO mice this is significantly impaired leading to increased IL-4 production and a non-protective Th2 response [27]. It is likely that in the Th2 biased response the monocyte derived cells recruited into these infiltrates in turn propel the nonprotective Th2 response, which would explain the differential effect of the CCR2-pathway interception in the protective versus nonprotective models of pulmonary cryptococcosis. In sum, DCs play an important role in the restimulation of T cells in the local lung environment, which is required for the control of cryptococcal infections.

### 2.5. Terminal Effector Cells

While DCs play a critical role in generating a protective T cells response, they can also serve as terminal effector cells, and are capable of killing cryptococcal cells. This cryptococcal killing was observed when murine BMDCs and human monocyte-derived DCs were incubated with *C. neoformans* and complement or opsonizing antibodies [76]. Studies revealed that the contents of the DC lysosome have fungicidal activity and can kill the fungus in vitro [76,78]. Several inhibitors of reactive oxygen species (ROS) demonstrate the reduction in this activity, but not a complete abrogation, suggesting that non-oxidative mechanisms also play a role [75]. One such mechanism is seen in the lysosomal enzyme cathepsin B which directly creates holes in the fungal cell wall which then leads to cryptococcal death by osmotic lysis [78]. The DC lysosome also contains additional enzymes and proteins that help kill or inhibit the growth of *C. neoformans,* including DC lysosomal proteins Coronin, HNE, NOSTRIN, MMP25, and MPO, although the mechanisms of their antifungal activity remains to be determined [13]. Although the lysosomal contents have antifungal activity, an important evasion strategy of *C. neoformans* is the induction of lysosomal damage, which favors its survival inside macrophages. Although lysosomal damage has not been reported in DCs, when macrophages are classically activated using IFN-γ, lysosomal damage is abrogated [13]. In addition, pDCs can kill *Cryptococcus* through the generation of ROS [33]. Inducible nitric oxide synthase (iNOS), a common method of antimicrobial control used by phagocytes, is not required for the pDCs to control of *Cryptococcus* [33]. Interestingly when human DCs encounter *C. gattii* they are completely capable of recognizing and killing these cells, even though they do not mature for antigen presentation purposes [100]. However, while individual DCs can kill *C. neoformans*, the infection fails to trigger a coordinated immune response. Following ex vivo interaction with *C. neoformans*, RNA-sequencing data show that the cDC2s significantly downregulated the expression of MHC-I, suggesting these cells may have problems with antigen presentation. These DCs also significantly upregulate CYP1B1, a cytochrome P450 enzyme, suggesting that metabolism plays a role in the antifungal activity (or lack thereof) in specific DC subsets [101].

### 2.6. Role of DCs in Immune Memory

DCs also have a major role in establishing a protective memory against cryptococcal infections via “trained immunity”. The mechanism of this DC memory response relies on epigenetic changes to the DCs, leading them to respond to *Cryptococcus* during later exposure [31,32,102,103]. The development of several experimental vaccines aided our understanding of vaccine-mediated protective immunity against cryptococcosis [104,105]. DCs isolated from mice and protectively immunized with H99γ led to increased IFN-γ and pro-inflammatory cytokine production ex vivo when incubated with *C. neoformans* [30]. In addition, this response indicated a memory-like DC response, mediated by epigenetic modification to several amino acids, that could help elicit protective immunity in immunocompromised individuals [31].

In addition, DC-based vaccines have received growing interest as a way to protect against *C. gattii* infections. Immunization studies reveal that DC-based vaccines improve responses against pulmonary infection by increasing the levels of IL-17A, TNF-α, and IFN-γ and producing lymphocytes during *C. gattii* infection [106]. Studies involving immunization confirm that Th1 and Th17 responses play an important role in vaccine-induced protection against highly virulent *C. gattii* [106]. BMDCs, when injected intravenously, migrate to lungs where they attract recipient MoDCs, leading to the stimulation of long-lived Th17 cells. Thus, systemic DC vaccines can induce lung-resident memory Th17 cells that helps stimulate immune responses against *C. gattii* [107].

## 3. DC Modulation by Cryptococcal Factors

No discussion of the role of DCs in infections would be complete without considering the ways in which pathogens try to evade these cells, and *Cryptococcus* is no exception. Several virulence factors in *Cryptococcus* have been identified that alter their interactions with DCs. Several of the major factors that contribute to DC–*Cryptococcus* interactions involve the fungal cell wall and capsule composition. As much has been written on this topic [108,109,110], this review will focus on capsule–DC interactions.

The capsule which surrounds the cryptococcal cell wall has been identified as a major player in how the fungal cells interact with the host DCs. *Cryptococcus* has large glucuronoxylomannan (GXM) polysaccharide capsules, which are usually phagocytosed by macrophages and DCs when shed during infection. However, the GXM can persist in the serum and CSF of patients even after antifungal therapy, thereby acting as a major virulence factor. GXM from *C. neoformans* acts to non-specifically inhibit T cell proliferation following antigen stimulation by DCs, leading to reduced T cell responses [111]. In *C. gattii*, GXM blocks extracellular receptor signaling dependent on TNF-α and p38MAPK that is required for the maturation of human-monocyte-derived DCs along with reducing the phagocytic process, which is important for DC maturation and antigen processing, respectively [112]. In addition, the capsule from *C. gattii* is better at suppressing the inflammatory cytokines from DCs than the *C. neoformans* capsule due to altered deacetylation of glucuronoxylomannan (GXM) in the *C. gattii* capsule [113].

Cryptococcal mannoprotein (MP), another soluble component of capsules, stimulates murine BMDCs to upregulate IL-10 and IL-10R, which skews them to a nonprotective DC type 2 activation [114]. Studies analyzing the interaction of DCs with encapsulated strains of *C. neoformans* reveal that the presence of a capsule is an obstacle to the maturation and activation of DCs and their subsequent stimulation of pro-inflammatory cytokines and induction of chemokine genes [115,116,117,118]. The inability of encapsulated strains to activate monocyte-derived DCs stimulated with GM-CSF and IL-4 indicated new pathways by which *C. neoformans* avoids efficient T cell responses [115]. However, not all acapsular mutants induce DC maturation, indicating further complexity in DC–cryptococcal interactions [119]. In total, the role of a capsule in avoiding DC maturation and helping hide the cryptococcal cells from detection and processing by DCs is well-established.

Regarding immune-modulatory cell-wall components, chitin received a lot of well-deserved attention. Chitin is a major component of the fungal cell wall and has a variety of impacts on the interactions of the fungi with various immune cells. Chitin abundance impacted cryptococcal-antigen-specific Th2 cell accumulation in the lungs, coordinated by resident cDC2 cells. This was dependent on chitotriosidase, a mammalian chitinase, cleaving fungal chitin during infection [93]. Another cell wall factor that aids in cryptococcal immune defenses is xylose. Using xylose-deficient mutants, researchers found that these cryptococcal cells lacking xylose had increased T-cell recruitment in murine models of infection which was driven by an increased pro-inflammatory response (IL-1β, IL-6, TNF-α, and IL-12p40) from cDC2 cells [120]. In vitro stimulation of BMDCs with xylose-deficient fungi resulted in increased levels of IL-1β, IL-6, TNF-α, and IL-12p40, similar to acapsular mutants [121]. Thus, xylose is an important mediator in cryptococcal–DC interactions.

Other additional cryptococcal virulence factors that aid in their avoidance of a protective DC response are abundant. Urease, which catalyzes the hydrolysis of urea to ammonia and carbon dioxide, is a well-known virulence factor in *Cryptococcus*. In a pulmonary murine model of infection, a urease deficient (*ure1*) mutant had reduced fungal loads and is required for the fungi to alter the host immune response to a non-protective Th2 response [120]. In addition, mice infected with the urease-deficient strain had reduced numbers of cDC2 in both the lung and lung-associated lymph nodes (LALN) by week twp compared to WT infections with reduced numbers and percentages of immature DCs in the LALN [120]. This suggests that cryptococcal urease production promotes a non-protective Th2 response via T cell stimulation by large numbers of cDC2 in the LALN, thereby linking urease to DC maturation [120].

Another major virulence factor for *C. neoformans* is the F-box protein 1 (Fbp1) [122,123]. FBP1 is a member of the ubiquitin ligase complex involved in proteasome degradation [124]. In a murine pulmonary model of infection, deletion of FBP1 resulted in increased recruitment of CCR2^+^ monocytes to the lungs and their rapid differentiation into DCs [125]. While the exact mechanism is unknown, FBP1 provides insight into how a fundamental cellular process in non-pathogenic organisms can have unpredictable effects on interactions with the immune system. Still, further work must be conducted to identify all of the ways in which *Cryptococcus* can alter DC interactions. For example, it has been shown that cryptococcal culture supernatants can suppress DC activation when DCs are activated by cryptococcal DNA through TLR9 signaling, but not when DCs are stimulated with DNA from other organisms [126]. The exact molecules and mechanism of this suppression remain unknown. In addition, the specific subset of DC that interacts with the organism may also influence this interaction based on the genes up- or downregulated in specific subsets. Thus, there are still many ways in which *Cryptococcus* interferes with DC responses that we have yet to uncover.

*C. gattii* is particularly capable of suppressing DC responses which is a major factor for its increased virulence. Several studies have begun to unravel the way in which *C. gattii* suppresses the immune system through its interactions with DCs. *C. gattii* infections have been shown to generate a reduced Th1 and Th17 response in mice pulmonary infections [28]. *C. gattii* reduces the ability of DCs to differentiate T cells in Th1 or Th17 cells via the reduced expression of co-stimulatory molecules and a lower expression of IL-12 and IL-23 by DCs [28]. Further work into the interactions of human DCs and *C. gattii* has shown that while cryptococcal cells can be efficiently phagocytosed and killed by human monocyte-derived DCs, these cells do not mature, leading to impaired T cell proliferation responses and immune dysregulation [100]. The DC paralysis that *C. gattii* induces is linked to the generation of an F-actin cage which prevents the maturation of DCs that phagocytose *C. gattii* cells [127]. However, additional studies are required to understand the mechanisms by which *C. gattii* can subvert DC-mediated immunity.

## 4. Conclusions

DCs are key players in anti-cryptococcal host defenses, serving multiple functions in the control of this fungal pathogen. As effective control of *Cryptococcus* depends on the quality of the T cell response, DCs are essential in inducing the protective immune response by performing APC functions: (a) antigen sensing; (b) phagocytosis and degradation of *C. neoformans* antigens; (c) presenting antigen to the T cells; (d) inducing antigen-specific T-cell proliferation; and (e) orchestrating T cell polarization. This occurs by the delivery of classical signals of antigen specific T cell activation and the production of immune polarizing cytokines. In addition, DCs promote effector T cell responses by providing antigen-specific restimulation at the site of infection to trigger the effector cytokine production. Finally, DCs act as distal effector cells, actively killing the ingested fungal cells and serving as a mechanism of direct fungal control, with emerging evidence of memory-type responses. While the importance of DCs in cryptococcal infections is well-established, identifying and characterizing the various subsets of DCs that carry out these functions is still a rapidly developing area. It is clear that DCs exist in multiple distinct subsets, each possibly playing a differential role in antifungal defenses. In addition, each of these subsets interact uniquely with specific fungal factors to lead to differential responses that either promote immunoprotection or fungal exploitation of the immune system to its own advantage. Thus, we predict that DCs will remain a center of focus in antifungal defense research and will be exploited for their potential to boost antifungal host responses and for the generation of antifungal vaccines.

## Figures and Tables

**Table 1:** Mouse DC subsets by Surface Markers.

DC Subset	Description	Location	Surface Markers	References
cDC2	CD11b^+^ Conventional Pulmonary DCs	Lungs	CD11c^+^/CD24^+^/CD11b^+^/CD172α^+^	[11,14,25]
cDC1	CD103^+^ Conventional Pulmonary DCs	Lungs	CD11c^+^/CD24^+^/CD11b^−^/CD103^+^	[11,14,25]
pDCs	Plasmacytoid DCs (pDCs)	Lungs and circulation	CD11c^int^/PDCA1^+^/B220^+^	[15]
MoDCs	Inflammatory monocyte-derived DCs (MoDCs)	Lungs, from circulation	CD11b^+^, CD11c^+^, F4/80^+^, Ly-6C^+^, MHC II^+^, and CD64^+^(Fc gamma RI^+^)	[25,26]
inf-cDC2	Inflammatory cDC	Lungs (only during inflammation)	CD26^+^, CD172A^+^,	[27]
LCs	Langerhans DCs	Epidermal layer of skin	CD11c^hi^, Langerin^+^ (CD207^+^), EpCam^+^, CLEC9a^−^, CD209^+^, DEC205^+^, F4/80^+^, CD103^−^, CD11b^+^, CD207^+^	[11]
LDC	Lymphoid DCs	Peripheral lymphoid organs	CD11c^+^ CD11b^−^ DEC205^hi^CD8^hi^	[16]
BMDCs	Bone marrow derived dendritic cells	Bone marrow	CD11c^+^ F4/80^−^	[17]

Murine dendritic cells are classified into several subsets, and the nomenclature used is dependent on the location and cell surface markers [11,14,15,16,17,25,28].

**Table 2:** Human DC subsets by Surface Markers.

DC Subset	Description	Location	Surface Markers	References
CD1c^+^ CD14^−^ DC	Conventional pulmonary DC subset	Lungs	BDCA1^+^(CD1c^+^)/CD14^−^/CD11c^+^	[21,23,25]
CD1c^+^ CD14^+^ DC	Conventional pulmonary DC subset	Lungs	BDCA1^+^(CD1c^+^)/ CD14^+^/CD11c^+^	[21,23,25]
Langerin^+^ DC	Langerin DCs	Lungs	HLA-DR^+^/langerin^+^	[21]
pDC	Plasmacytoid DCs (pDCs)	Lungs	BDCA2^+^(CD303^+^)/ CD11c^−^/CD123^+^	[23]
DC precursor	Classical monocytes	Circulation	CD14^hi^ CD16^−^	[20]
DC precursor	Non-classical monocytes	Circulation	CD16^hi^ CD14^lo^	[20]
DC precursor	Intermediate monocytes	Circulation	CD14^hi^ CD16^hi^	[20]

Human dendritic cells are classified into several subsets, and the nomenclature used is dependent on the location and cell surface markers [20,21,23,25].
